# Extended RF shimming: Sequence‐level parallel transmission optimization applied to steady‐state free precession MRI of the heart

**DOI:** 10.1002/nbm.3701

**Published:** 2017-02-14

**Authors:** Arian Beqiri, Anthony N. Price, Francesco Padormo, Joseph V. Hajnal, Shaihan J. Malik

**Affiliations:** ^1^ Division of Imaging Sciences and Biomedical EngineeringKing's College LondonLondonUK; ^2^ Centre for the Developing BrainKing's College LondonLondonUK

**Keywords:** cardiac, parallel transmission MRI, RF shimming, SAR

## Abstract

Cardiac magnetic resonance imaging (MRI) at high field presents challenges because of the high specific absorption rate and significant transmit field (*B*_1_^+^) inhomogeneities. Parallel transmission MRI offers the ability to correct for both issues at the level of individual radiofrequency (RF) pulses, but must operate within strict hardware and safety constraints. The constraints are themselves affected by sequence parameters, such as the RF pulse duration and TR, meaning that an overall optimal operating point exists for a given sequence. This work seeks to obtain optimal performance by performing a ‘sequence‐level’ optimization in which pulse sequence parameters are included as part of an RF shimming calculation. The method is applied to balanced steady‐state free precession cardiac MRI with the objective of minimizing TR, hence reducing the imaging duration. Results are demonstrated using an eight‐channel parallel transmit system operating at 3 T, with an *in vivo* study carried out on seven male subjects of varying body mass index (BMI). Compared with single‐channel operation, a mean‐squared‐error shimming approach leads to reduced imaging durations of 32 ± 3% with simultaneous improvement in flip angle homogeneity of 32 ± 8% within the myocardium.

**Abbreviations used**
BMI: body mass index
bSSFP: balanced steady‐state free precession
CNR: contrast‐to‐noise ratio
DREAM: dual refocusing echo acquisition mode
EM: electromagnetic
IEC: International Electrotechnical Commission
MRI: magnetic resonance imaging
MSE: minimum squared error
PTx: parallel transmission
RF: radiofrequency
ROI: region of interest
SAR: specific absorption rate
SNR: signal‐to‐noise ratio
TEM: transverse electromagnetic
UHF: ultra‐high field
VCG: vector cardiograph
VOP: virtual observation point

## 1 INTRODUCTION

High‐field (≥3 T) cardiac magnetic resonance imaging (MRI) offers considerable gains in signal‐to‐noise ratio (SNR) and improved blood–tissue contrast, provided the optimum flip angle can be realized.^1^ However, these gains come with a number of challenges, primarily due to the altered electromagnetic (EM) conditions when imaging at high field. Transmit field inhomogeneity is caused by increasing EM interaction between the subject and the radiofrequency (RF) transmit coil at higher Larmor frequencies. Furthermore, higher specific absorption rate (SAR) is induced in subjects than at lower field, but the same regulatory limits must be adhered to regardless of field strength. Balanced steady‐state free precession (bSSFP) sequences are commonly used in cardiac MRI, but constraining operation to be within maximum regulatory SAR limits^2^ can present a serious limitation. An improvement in SAR efficiency, i.e. the ability to achieve current imaging protocols at a reduced SAR level or to achieve improved imaging within the maximum SAR limits, would lead to numerous benefits. Flip angles could be increased to improve contrast, and TRs could be decreased to reduce banding artifacts^3^ and to shorten scan times. Shorter scan times would be greatly beneficial as patient breath‐holds could be reduced accordingly. Both transmit field inhomogeneity and SAR levels are subject dependent so a generic solution cannot be applied that would account for these in every scenario — a more tailored methodology is required. Defining such a methodology for optimally efficient performance on a subject‐specific basis for bSSFP cardiac MRI is the key aim of this work.

Parallel transmission (PTx) MRI uses multiple independent channels to generate RF fields. ‘RF shimming’^4^ can then be used to control both magnetic (*B*_1_^+^) and electric components of the RF fields, by adjusting the relative weighting applied to each channel, often in a subject‐specific way. RF shimming is generally used to improve the homogeneity of *B*_1_^+^ and has been demonstrated previously for cardiac imaging at 3 T using a two‐channel, clinical PTx MRI system.^5^, ^6^ However, RF shimming can also be used to control SAR^7^ by performing a constrained optimization subject to a strict set of SAR and system power constraints.^8^ A number of groups have demonstrated the efficacy of SAR‐constrained RF shimming in simulation.^7^, ^9^, ^10^, ^11^

Further constraints commonly encountered when using transmit arrays are peak forward power and average power limits from the RF amplifiers.^8^, ^9^, ^12^ For the hardware used in this study (and in many reports in the literature^12^, ^13^), these limits are easily reached under standard operation during body imaging. Current RF shimming approaches concentrate on the homogeneity of the achieved *B*_1_^+^ field independently from the sequence into which the excitation pulse is embedded. This is an issue because the hardware and safety constraints on the RF shimming calculation are dependent on the properties of the sequence itself. In this work, we explore an extension, which we refer to as ‘sequence‐level PTx optimization’, in which the sequence parameters (RF pulse duration, TR, etc.) are optimized in conjunction with RF shim settings in order to achieve some overall objective. We focus on bSSFP sequences typically used for cardiac MRI with the overall objective of minimizing TR, which has the advantage of reducing breath‐hold durations and banding artifacts. 3 T cardiac imaging using an eight‐channel PTx coil is used to demonstrate the method.

## 2 METHODS

### 2.1 Sequence‐level PTx optimization framework

In a typical MRI system architecture, the production of an RF pulse begins with a low‐level RF waveform *p*(*t*) which i*s* amplified and fed to the coil, which produces a pulsed *B*_1_^+^ field of a certain amplitude (typically in the μT range) within the object to be imaged. In this work, we treat *p*(*t*) directly in units of μT and note that there is a hidden scaling factor between the field produced and the voltage signal on the RF generator that can be made explicit if necessary. RF inhomogeneity and subject‐specific loading effects mean that the true *B*_1_^+^ field may become spatially variable; hence, we introduce a dimensionless scaling factor *S*(***r***), referred to here as the transmit sensitivity of the RF coil:

(1) $$B_{1}^{+}\left(r,t\right)=S\left(r\right)p\left(t\right)$$

*S*(***r***) can deviate from the ideal value of unity because of inhomogeneity effects at high RF frequencies, but also due to loading changing the efficiency of the coil. The flip angle, which is also generally spatially varying, is then defined by the integral:

(2) $$\theta \left(r\right)=\gamma \int_{0}^{\tau }B_{1}^{+}\left(r,t\right)\mathrm{dt}=\mathrm{\gamma S}\left(r\right)\int_{0}^{\tau }p\left(t\right)\mathrm{dt}=\delta_{1}\;p_{\max}\;\tau \;\gamma \;S\left(r\right)$$

where *γ* is the gyromagnetic ratio (rad/μT/s), *τ* is the pulse duration and *δ*_1_ is the relative duration of a block pulse that generates the same flip angle with the same peak amplitude *p*_max_ ≡ max{|*p*(*t*)|} given as:

(3) $$\delta_{1}\equiv \frac{\int_{0}^{\tau }p\left(t\right)\mathrm{dt}}{p_{\max}\;\tau }$$

It should be noted that for simple excitation pulses of the type generally used with bSSFP sequences, Equation (2) gives the flip angle at the center of the slice and is not limited to low flip angles; we ignore the effect of changing slice profile as the flip angle changes.

For a multi‐channel transmit system, the total *B*_1_^+^ field produced is given by a linear superposition of fields from each channel. We introduce a vector ***w*** of complex channel‐specific weightings referred to as RF shims; the total *B*_1_^+^ is the weighted sum:

(4) $$B_{1\;\mathrm{tot}}^{+}\left(r,t\right)=\sum_{i=1}^{N_{c}}w_{i}B_{1\;i}^{+}\left(r,t\right)$$

Similarly, the flip angle is a linear sum over all transmit channels, which may be written as:

(5) $$\theta \left(r\right)=\delta_{1}\;p_{\max}\;\tau \;\gamma \;\sum_{j=1}^{\mathrm{Nc}}S_{j}\left(r\right)w_{j}\;$$

where *S*_*j*_(***r***) is the sensitivity of the *j*th channel, usually measured using a *B*_1_^+^ map. This may further be written as a matrix–vector product:

(6) $$\theta =\delta_{1}\;p_{\max}\;\tau \;\gamma \;\mathrm{Sw}=S_{\theta }w\;$$

where ***S*** is a matrix of the acquired transmit sensitivities for all channels (number of voxels × number of channels), ***w*** is a column vector of complex channel‐specific weighting factors and ***θ*** is a vector of achieved flip angles (length is the number of voxels). In order to simplify the expressions, we define ***S***_***θ***_ ≡ *θ*_0_ ***S*** as the sensitivity of the system in units of flip angle, which directly relates the achieved flip angle to the input weighting factors ***w*** for a given pulse *p*(*t*). *θ*_0_ ≡ *δ*_*1*_ *p*_max_ *τγ* is the flip angle that would be achieved by waveform *p*(*t*) at unit sensitivity.

#### 2.1.1 Hardware constraints

The peak and average power provided by the RF amplifiers are related to the peak RF pulse amplitude as:

(7) $$\begin{array}{l} \text{peak power}=p_{\max}^{2}A\\ \text{average power}=p_{\max}^{2}A\Delta \end{array}$$

where *A* (W/μT^2^) is a scaling constant related to the efficiency of the RF chain. Δ is the power duty cycle of the sequence:

(8) $$\Delta \equiv \frac{\delta_{2}\;\tau }{\mathrm{TR}}\;$$

TR is the repetition time and *δ*_2_ is the relative energy of a block pulse scaled to have the same flip angle and maximum amplitude as *p*(*t*):

(9) $$\delta_{2}\equiv \frac{\int_{0}^{\tau }p^{2}\left(t\right)\mathrm{dt}}{p_{\max}^{2}\;\tau }$$

As with *δ*_1_, *δ*_2_ is an intrinsic property of the RF pulse shape used. RF shims ***w*** act as a multiplier of the RF waveforms; hence the peak forward power limit *P*_peak_ on each channel (assumed to be equal) can be translated into a limit on the applied RF shims as:

(10) $$\;\left|w_{j}\right|\leq \frac{1}{p_{\max}}\sqrt{\frac{P_{\text{peak}}}{A}}\;\forall j$$

and the average power constraint (per channel) *P*_*av*_ as:

(11) $$\left|w_{j}\right|\leq \frac{1}{p_{\max}}\sqrt{\frac{P_{\text{av}}}{A\Delta }}\;\forall j$$

**Figure 1:**
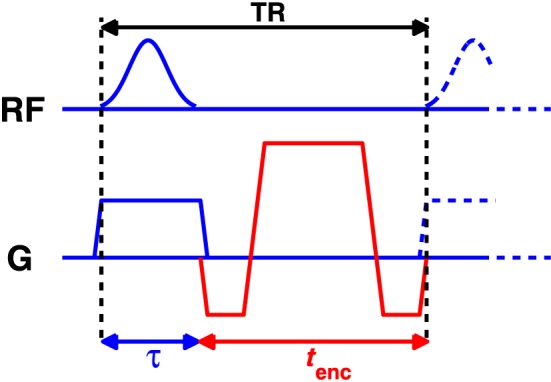
Figure 1 Timing diagram of balanced steady‐state free precession (bSSFP). The overall sequence TR must be sufficiently long to include the radiofrequency (RF) pulse and the spatial encoding gradients. The RF amplifier gating duty cycle limits also constrain the pulse duration relative to the TR period

A final hardware constraint is the RF amplifier gating duty cycle limit – a limit on the fractional amount of time for which the amplifier can operate irrespective of the power demand. For the amplifiers used in this work, the value is 50%. This, together with the need to physically fit both the RF pulse and spatial encoding gradients into each TR period, gives a relation for the minimum achievable TR irrespective of power or safety limits:

(12) $${\mathrm{TR}}_{\min}=\max\left\{\begin{array}{c} \tau +t_{\mathrm{enc}}\\ \tau /\delta_{0} \end{array}\right\}$$

where *t*_enc_ is the time required for spatial encoding (see Figure 1) and *δ*_0_ is the gating duty cycle limit (*δ*_0_ = 0.5).

#### 2.1.2 SAR constraints

SAR estimates were obtained from an EM model of the coil loaded with a suitable human model (details are provided later). The resulting fields were used to compute local 10 g averaged *Q*‐matrices^14^, ^15^ from which SAR for any set of RF shims ***w*** can be obtained from the *Q*‐matrices by the evaluation of w*Qw (* indicates Hermitian transpose).^14^ A single whole‐body *Q*‐matrix was also constructed from which whole‐body SAR can be evaluated. The set of *Q*‐matrices was compressed using the virtual observation points (VOPs) method^16^; two levels of compression were used, as discussed later.

A key issue is normalization of the EM model to match scanning conditions. There are many possible approaches for normalization, and the method used must be appropriate for the type of transmit coil used, which in this work, was an eight‐channel, whole‐body transverse electromagnetic (TEM) array (described in detail in Vernickel et al^17^). The array is built into the bore of the scanner, with elements distributed around the subject, and has a well‐defined ‘quadrature’ (birdcage‐like) setting in which the elements are driven with equal amplitude, but 45° phase increments, to give a nominally circularly polarized field, summarized by the RF shim vector ***w***_**quad**_:

(13) $$w_{\text{quad},j}=e^{\frac{\mathrm{i\pi}\left(j-1\right)}{4}}\;j=1,2,\ldots ,8$$

The EM fields obtained from the model were normalized such that application of ***w***_**quad**_ results in an excitation with mean *B*_1_^+^ = 1 μT in the imaging slice; *Q*‐matrix elements have units of W/kg/μT. The maximum local SAR (*lSAR_max_*) can thus be evaluated as:

(14) $${\text{lSAR}}_{\max}=\max_{i}\left\{w^{*}Q_{i}w\right\}\times {\left(B_{1\;\text{achieved}}^{+}\right)}^{2}\times \Delta$$

where *i* is an index over the (compressed) set of *Q*‐matrices. *B*_1_^+^_achieved_ is the mean *B*_1_^+^ field in a slice measured experimentally in quadrature mode:

(15) $$B_{1\;\text{achieved}}^{+}={\;p}_{\max}\bar{S\;w_{\text{quad}}}$$

where the overbar indicates a spatial average over the imaging slice. This scaling factor is used to match scanning conditions to the EM model. The normalization used here is appropriate for an enveloping ‘body’‐type coil with a naturally defined ‘quadrature’ mode; however in principle other equivalent measurements between the simulation and real world experiment could be used.

#### 2.1.3 Constrained optimization, PTx case

The overall aim of the optimization explored in this article is to minimize TR subject to the appropriate hardware and safety constraints, and the mean flip angle within the region of interest (ROI) being equal to the target flip angle *θ*_0_. This may be written as:

(16) $$\begin{array}{l} \arg\;\min_{w,\tau }\;\left\{\mathrm{TR}\right\}\\ s.t.\;{\text{constraints}}_{w}\left(\tau \right)\\ {\bar{\theta }}_{\mathrm{ROI}}=\theta_{0} \end{array}$$

where ${\bar{\theta }}_{\mathrm{ROI}}$ is the mean flip angle within an ROI encompassing the myocardium. The constraints apply to the values of ***w***, but are themselves functions of the pulse duration *τ* and TR:

(17) constraintswτ:maxiw*Qiw×Swquad¯2×θ02γ2×δ2δ12×1τTR≤lSARmaxw*Qwbw×Swquad¯2×θ02γ2×δ2δ12×1τTR≤wbSARmaxwj≤τPpeakAδ1γθ0∀jwj≤τTRδ1γθ0δ2PavA∀j

*lSAR_max_* and *wbSAR_max_* are the maximum local and whole‐body SAR constraints, taken to be the International Electrotechnical Commission (IEC) normal mode limits of 10 W/kg and 2 W/kg, respectively.^2^ Note that for given *τ*, the minimum TR is defined by Equation (12), and hence the constraints are written purely as functions of *τ*.

The overall optimization defined in Equation (16) is performed using a nested approach, with an outer step that optimizes the pulse duration (hence TR) and an inner step that optimizes RF shims ***w***, given the constraints for this specific pulse duration. The inner optimization may be formulated as a classic RF shimming problem. In this work, improvement in flip angle homogeneity is not a specific aim of the optimization; instead we only wish to minimize TR subject to the mean flip angle being equal to the target. We propose two alternative versions for the inner optimization. The first is to minimize the mean bias in the flip angle:

(18) $$\begin{array}{l} {\arg\min}_{w}∥\bar{\left|S_{\theta }w\right|}-\theta_{0}{∥}_{\mathrm{ROI}}^{2}\\ s.t.\;{\text{constraints}}_{w}\left(\tau \right) \end{array}$$

The bias is the difference between the mean achieved flip angle and the target; hence a zero bias solution is optimal. This optimization has the drawback of not constraining the variance of the flip angle within the ROI, potentially resulting in a highly inhomogeneous flip angle within the target region (myocardium). Hence the second proposed inner optimization is to minimize the squared difference between the achieved flip angle and target within the ROI:

(19) $$\begin{array}{l} {\arg\min}_{w}||\;|S_{\theta }w|-\theta_{0}{∥}_{\mathrm{ROI}}^{2}\\ s.t.\;{\text{constraints}}_{w}\left(\tau \right) \end{array}$$

Since the mean squared error can be expressed as the sum of the variance and the square of the bias,^18^ optimal solutions for this second minimization will jointly minimize bias and variance of the flip angle within the ROI, but will not necessarily have zero bias. The first optimization is referred to as ‘minimum bias’ and the second as ‘minimum squared error’ (MSE).

Neither of the inner optimizations is constrained to produce solutions with zero bias (i.e. ${\bar{\theta }}_{\mathrm{ROI}}=\theta_{0}$); instead, bias is constrained by using a penalty function in the outer optimization:

(20) $${\arg\min}_{\tau }\;\left\{\mathrm{TR}+f\left(\hat{\theta }\right)\right\}$$

where $\hat{\theta }\;$ is the flip angle bias and $f\left(\hat{\theta }\right)$ is a function designed to penalize high bias solutions. The bias is defined in percentage units:

(21) $$\hat{\theta }\equiv \frac{{\bar{\theta }}_{\mathrm{ROI}}-\theta_{0}}{\theta_{0}}\times 100$$

The outer optimization is not constrained; instead each evaluation of the cost function for a candidate pulse duration *τ* results in a constrained inner optimization (using either Equation (18) or Equation (19)), which will yield some optimal RF shims ***w*** with an associated flip angle bias $\hat{\theta }$. The overall cost of this solution in the outer optimization is the sum of the achieved minimum TR (from Equation (12)) and the penalty function. For the minimum bias optimization, the penalty function is defined straightforwardly as:

(22) $$f\left(\hat{\theta }\right)={\hat{\theta }}^{2}$$

For the MSE optimization, minimization of the squared error leads to a small, but non‐zero bias; hence we choose to accept a small, but non‐zero, flip angle bias in this case. In this work we chose an acceptable bias of 5% and defined the penalty function as:

(23) fθ^=θ^20θ^>5θ^≤5

which penalizes solutions with bias over 5%.

#### 2.1.4 Constrained optimization, quadrature case

**Figure 2:**
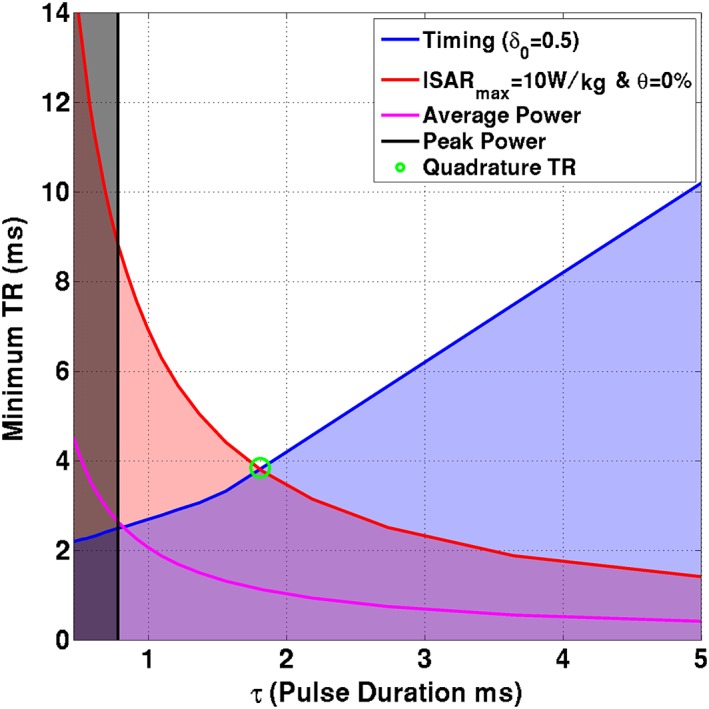
Constraints for quadrature case for one *in vivo* subject. Constraints are as indicated in the legend; shaded areas violate one or more constraints. Solutions along the blue line (minimum TR for given pulse duration) are sought by the optimization

The same procedure may be followed to determine the optimal operating point for a standard (non‐PTx) MRI system. This can be approximated with a PTx system by setting ***w*** *= d* ***w***_**quad**_, where ***w***_**quad**_ is a birdcage‐like mode of the transmit coil, as described above. With a single‐channel system, we can only vary the scaling parameter *d* and cannot affect the flip angle homogeneity within the ROI. Optimized single‐channel solutions are generated by performing the minimum bias optimization outlined above. The different constraints can be visualized straightforwardly for the quadrature case, as plotted in Figure 2. The blue line depicts the minimum TR for a given pulse duration (Equation (12)) – we seek a solution on this line. The red line represents minimum TR for each *τ* when maximum local SAR is 10 W/kg and the bias is 0%. The black line indicates the peak power constraint – this is independent of TR and effectively sets a minimum pulse duration. The magenta line depicts the average power constraint. The optimum solution for quadrature (single‐channel) operation is given by the green circle. PTx optimization allows us to move further down the blue curve by producing lower SAR solutions, hence shifting the *lSAR_max_* curve. It should be noted that in the PTx case the solution is not easily depicted on a diagram of this type as the solution for each individual transmit channel can approach the hardware limits separately, and peak and average power limits become more important.

### 2.2 Experimental methods

Research ethics committee approval was obtained for the study; all participants gave written informed consent prior to enrolment. Experiments were performed on a Philips (Best, The Netherlands) Achieva 3 T system fitted with an in‐built eight‐channel TEM body coil, which replaces the standard birdcage coil in this scanner. The TEM coil is of a similar size to a standard birdcage (element lengths, 42 cm); a detailed description of the design of this coil is given by Vernickel et al.^17^ The scanner can control the relative phase and amplitude of each transmit element independently to perform RF shimming. It can also operate in nominal quadrature mode (i.e. a circularly polarized birdcage‐like mode, as described above) in which phase offsets between each element are fixed. Quadrature operation produces similar results to more standard birdcage RF coils.^19^ A bank of eight Analogic (Peabody, MA, USA) AN8134 RF amplifiers is used with the coil. The peak forward power and average power limits are set to 1 kW and 100 W per channel, respectively, measured at the scanner filter panel, and the scaling parameter *A* = 2.5 W/μT^2^. A six‐channel cardiac receive coil was used for signal reception.

#### 2.2.1 EM simulations

**Figure 3:**
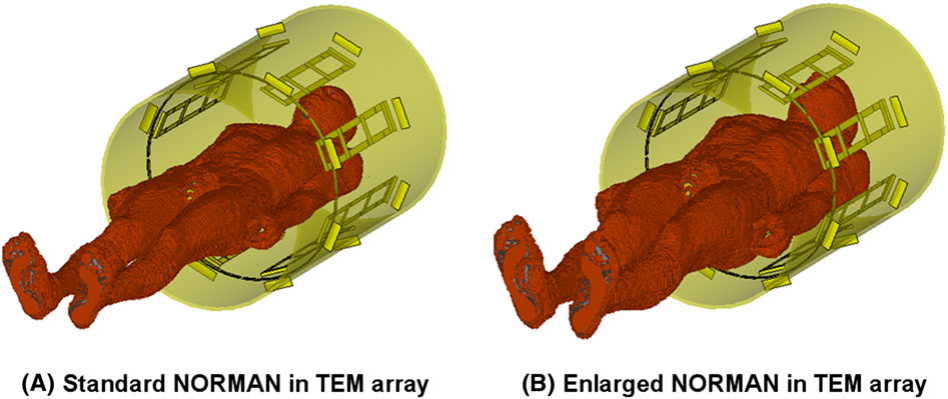
Eight‐channel transverse electromagnetic (TEM) array model with the standard NORMAN voxel model (A) and an enlarged version of the NORMAN model (B)

The body transmit array^17^ was modeled using the time‐domain Finite Integration Technique^20^ of CST Microwave Studio (CST AG, Darmstadt, Germany). The coil model was comprised of conductive elements modeled as lossy copper metal and all lumped elements were replaced with 50 Ω sources. This enabled the model to be tuned, matched and de‐coupled using circuit co‐simulation,^21^ which was implemented in Matlab (The Mathworks, Natick, MA, USA); the application of this method to our specific transmit coil is described in detail in Beqiri et al.^22^ The NORMAN voxel model,^23^ which has a BMI of 23.5, was placed heart‐centered in the coil (Figure 3A) for this tuning and matching process, in a similar manner to that performed in the physical coil.^17^ Another simulation was produced using the same coil model with an enlarged version of the NORMAN voxel model (Figure 3B), which was stretched in the anterior–posterior and left–right directions in order to emulate a larger male^24^ with a BMI of 31.

#### 2.2.2 Imaging experiments

RF shimming was performed for a bSSFP CINE sequence with the following parameters: flip angle = 45°; bandwidth = ~2.7 kHz (read‐out duration = ~0.37 ms); *t*_enc_ = 1.7 ms; resolution = 1.67 mm × 2 mm; slice thickness = 8 mm. Single‐slice images were acquired in the four‐chamber view of the heart. A flip angle of 45° was used as this was recommended for optimal blood tissue contrast in Schär et al.^25^ Scans were performed within single breath‐holds with duration depending on the field of view, the subjects' heart rates and the minimum achieved TRs, and were retrospectively gated to produce 30 heart phases. The excitation pulse was a Gaussian pulse with *δ*_1_ = 0.53 and *δ*_2_ = 0.40. Second‐order *B*_0_ shimming was performed using a *B*_0_ map‐based method similar to that described in Schär et al.^25^ A *B*_0_ map (cardiac gated with gate delay of 300 ms, 3 mm × 3.7 mm resolution, TE = 2.3 ms, ΔTE = 2.3 ms and TR = 5.8 ms) was acquired in the four‐chamber orientation, and the center frequency for bSSFP was set manually.

Seven male subjects were scanned in total and were matched to the corresponding SAR model according to their BMI. The first five subjects had an average mass of 73 kg (BMI = 22 ± 2.2 kg/m^2^) and were matched to the smaller NORMAN model. Subjects 6 and 7 had BMI values of 29 and 31 kg/m^2^, respectively, and were matched to the larger NORMAN model.

*B*_1_^+^ mapping used the DREAM method (dual refocusing echo acquisition mode^26^) because of its speed. Per‐channel *B*_1_^+^ maps were acquired for the same slice as the SSFP imaging. DREAM uses a magnetization‐prepared rapid gradient echo read‐out to acquire a single‐slice *B*_1_^+^ map in a single shot, allowing all channels to be measured in a breath‐hold with a duration of 11 s. The DREAM sequence is sensitive to flow and will give unreliable *B*_1_^+^ estimates in the blood pool for cardiac applications. The sequence was triggered to mid‐diastole to minimize flow effects, and manually drawn ROIs were used to exclude the blood pool and include only myocardium on the *B*_1_^+^ maps. The DREAM imaging parameters were as follows: imaging flip angle = 15°; pre‐pulse flip angle = 60°; resolution = 7 mm × 7 mm; slice thickness = 8 mm. Cardiac triggering used a four‐lead vector cardiograph (VCG) with a 0.5 s additional delay added between each individual DREAM acquisition to lessen saturation effects and ensure a maximum of one acquisition per heartbeat. Mapping was performed using linear combinations of channels to improve SNR.^27^

#### 2.2.3 Numerical optimizations

Computations were performed using Matlab 2014 (The Mathworks) on a Dell Precision T5600 Workstation (Round Rock, TX, USA). The outer unconstrained optimization (Equation (20)) was performed using MATLAB's *fminsearch* function. Each evaluation of the cost function requires solution of the inner optimization; *fminsearch* was chosen for this task because the simplex search algorithm^28^ it employs does not compute cost function derivatives, hence it uses relatively few function evaluations. Both forms of constrained inner optimization (Equations (18) and (19)) were solved using the CVX convex programming interface^29^, ^30^ with the SeDuMi^31^ solver. As formulated in Equations (18) and (19), both are not convex since they require evaluation of the magnitude of the flip angle distribution |***S***_***θ***_***w***|. The MSE inner optimization (Equation (19)) was solved as a ‘magnitude least squares’^32^ problem and approached using the variable exchange method.^33^ This method seeks to simultaneously optimize the RF shims ***w*** and an image phase *ϕ* via the complex variable ***z*** = exp(*iϕ*):

(24) $$\begin{array}{l} {\arg\min}_{w}∥{\;S}_{\theta }w-\theta_{0}{}^{\circ}z{∥}_{\mathrm{ROI}}^{2}\\ s.t.\;{\text{constraints}}_{w}\left(\tau \right) \end{array}$$

where ‘°’ indicates an element‐wise product. The image phase *ϕ* is initialized as the phase of a quadrature‐mode excitation, and then updated to the phase of the current solution at each iteration of the algorithm until convergence is reached.^32^ The minimum bias optimization (Equation (18)) was approached in a similar way by removing the projected image phase prior to calculation of the mean:

(25) $$\begin{array}{l} {\arg\min}_{w}∥\bar{\left(S_{\theta }w\right){\;{}^{\circ}z}^{*}}-\theta_{0}{∥}_{\mathrm{ROI}}^{2}\\ s.t.\;{\text{constraints}}_{w}\left(\tau \right) \end{array}$$

where the asterisk denotes complex conjugate. Constrained optimizations for these types of problems are inherently time intensive as all the constraints must be evaluated at each optimization step.^34^ In order to speed up the optimization run time, a number of modifications were made. A set of approximately 800 VOPs (3% overestimate bound) was used for the evaluation of the constraints during each optimization; reported local SAR values for solutions were then calculated with a 1% overestimate set for improved accuracy (approximately 3500 VOPs). This modification allows each CVX optimization to take 2–3 s. The ‘variable exchange’ methods above were modified by only computing the optimal ***z*** once (i.e. for the first iteration of the outer optimization); all subsequent iterations used the same ***z***. As 10–20 iterations are often required to find an optimal ***z***, this represents a large speed‐up. The outer optimization converged within 10 iterations. For all seven subjects, minimum bias and MSE optimizations were performed, and optimal quadrature mode settings were also computed for comparison; imaging using all three sequences was performed. The MSE optimization was completed within approximately 5 min; the minimum bias optimization was faster (approximately 3 min); optimized quadrature (single‐channel) solutions were computed within milliseconds.

#### 2.2.4 Data and code availability

Matlab code, together with an example *in vivo B*_1_^+^ dataset, and relevant data from EM simulations necessary to reproduce the presented calculations used in the *in vivo* experiments, are available at https://github.com/mriphysics/cardiac_RF_shimming.

## 3 RESULTS

**Figure 4:**
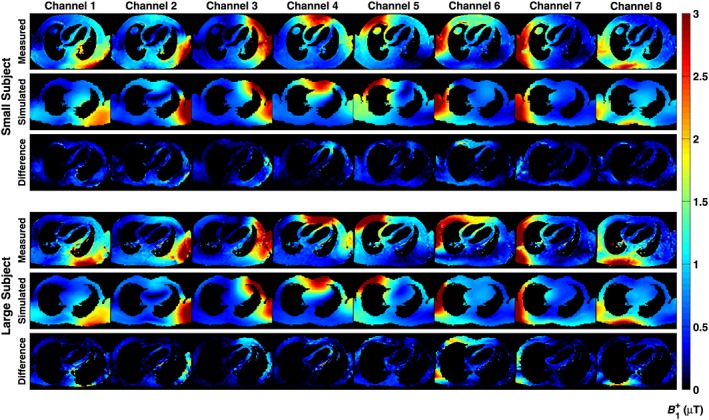
Comparison between measurement and simulation for the *B*_1_^+^ fields in single‐slice, four‐chamber views through the heart for a single small and single large subject, with the relevant electromagnetic (EM) model. Subtraction images are shown below each case; note that the simulated and measured maps do not precisely co‐align and voxels not present in both maps are excluded from the subtraction

Figure 4 compares simulated *B*_1_^+^ fields in a four‐chamber view of the heart from both voxel models with those measured in matched subjects *in vivo*. The four‐chamber view is not aligned with the symmetry axis of the coil, so the *B*_1_^+^ maps appear to be left–right asymmetric. The equivalent oblique view was extracted from the simulations for comparison, and there is qualitative agreement between these. As the subject and model differ in detail, the subtraction images are masked to only show pixels that are present in the overlap between the two maps. Quantitatively, the mean correlation coefficient between acquired and simulated *B*_1_^+^ maps over all subjects and transmit channels was 0.84 ± 0.04.

**Figure 5:**
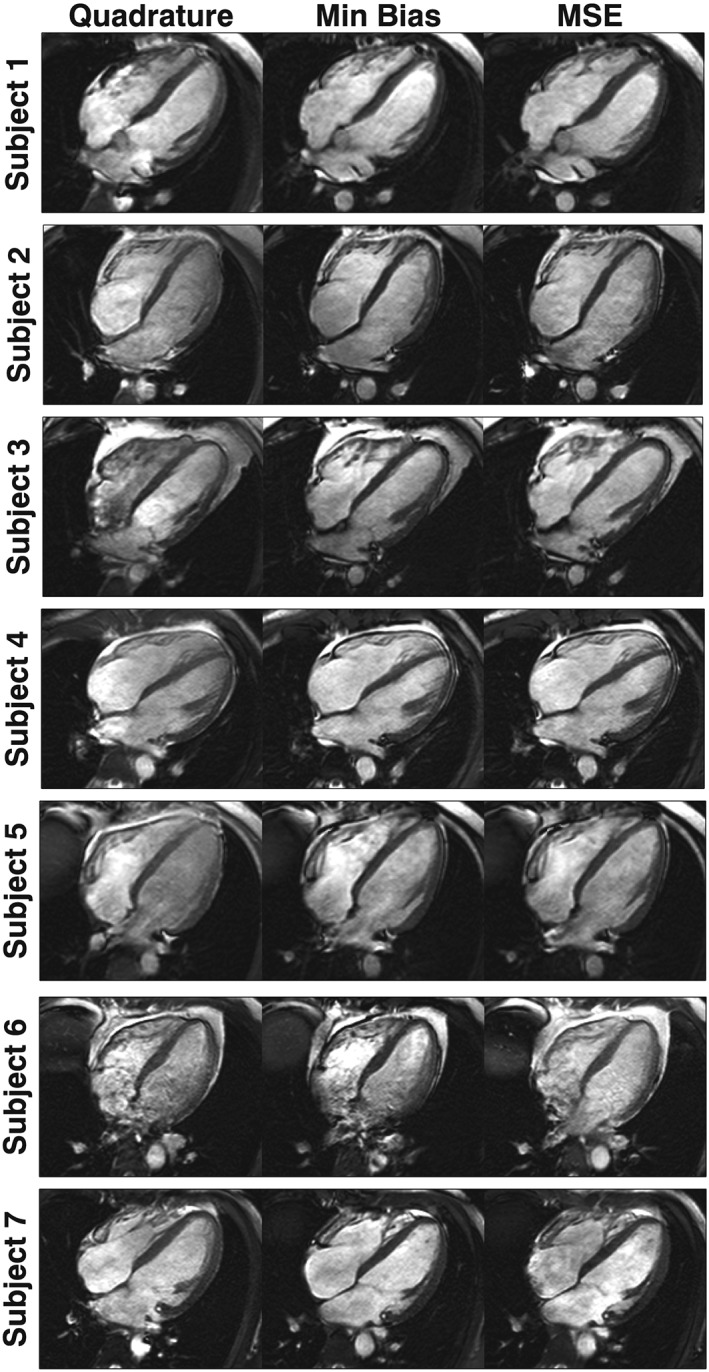
Balanced steady‐state free precession (bSSFP) imaging data shown for all subjects for quadrature, minimum bias and minimum squared error (MSE) shimming. Matched cardiac phases are shown at end diastole

**Figure 6:**
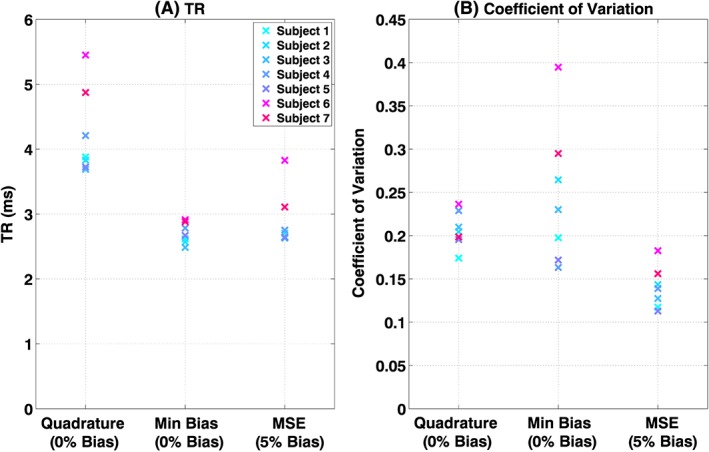
TR (A) and coefficient of variation (B) in *B*_1_^+^ shown for quadrature, minimum bias and minimum squared error (MSE) shimmed solutions. All sequences had *lSAR_max_* =10 W/kg

Figure 5 shows the *in vivo* bSSFP imaging data acquired from all subjects, with all three imaging scenarios (quadrature, minimum bias and MSE). Figure S1 shows boxplots of the contrast‐to‐noise ratio (CNR) between the myocardium and blood pool within the heart for all the images. Figure 6 shows the TR for each sequence and the coefficient of variation of *B*_1_^+^ measured in the heart. The PTx optimized sequences had shorter TRs; minimum bias shim led to a reduction in TR of 36 ± 6% compared with the optimized quadrature sequence, whereas the MSE shim led to a TR reduction of 32 ± 3%. The effect of the reduced TRs was to reduce breath‐hold durations by the same amount from 11 to 15 s to 6.5–9 s depending on the subject.

**Figure 7:**
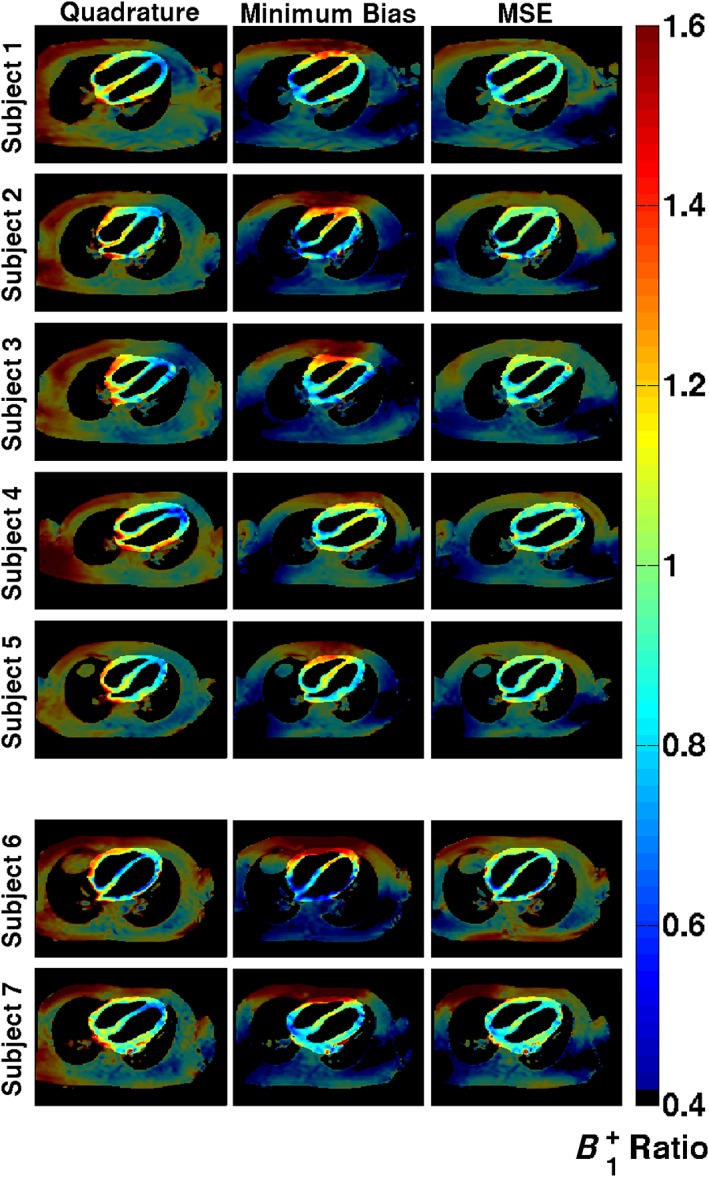
*B*_1_^+^ maps in the four‐chamber view shown for all subjects for quadrature, minimum bias and minimum squared error (MSE) shimming. Each map is normalized to the desired *B*_1_^+^ value, so a value of 1.0 is ideal. The region of interest used for optimization is highlighted in each case

**Figure 8:**
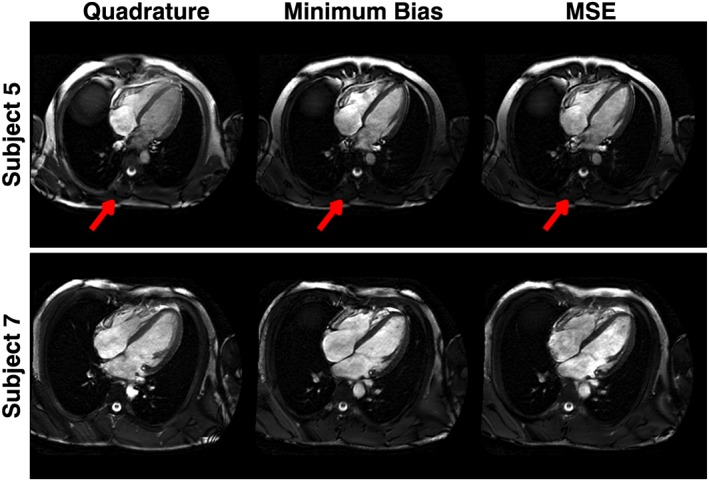
Full field‐of‐view images for two subjects. The radiofrequency (RF) shimmed solutions (both minimum bias and minimum squared error, MSE) result in low *B*_1_^+^ in the posterior part of the torso, resulting in low signal (arrows). The image quality for the heart is uncompromised

Figure 7 shows the measured *in vivo B*_1_^+^ maps for each of the imaged scenarios. In quadrature mode, the *B*_1_^+^ field is quite variable within the heart – for example in all five smaller subjects, there is a drop in *B*_1_^+^ from the base to the apex of the heart. The mean coefficient of variation is 0.21 (21% variation) for quadrature operation. The minimum bias shim leads to a worsening in homogeneity for some subjects. The MSE shim leads to an improvement in homogeneity in all cases (average of 33 ± 8%). Consequently, image quality for the MSE shim appears to be most consistent. The local nature of the shimming optimization leads to inhomogeneous *B*_1_^+^ in areas outside the heart, as visible in Figure 7. Figure 8 shows two selected full field‐of‐view images demonstrating that the effect does alter contrast particularly in posterior regions, but does not affect the heart.

**Figure 9:**
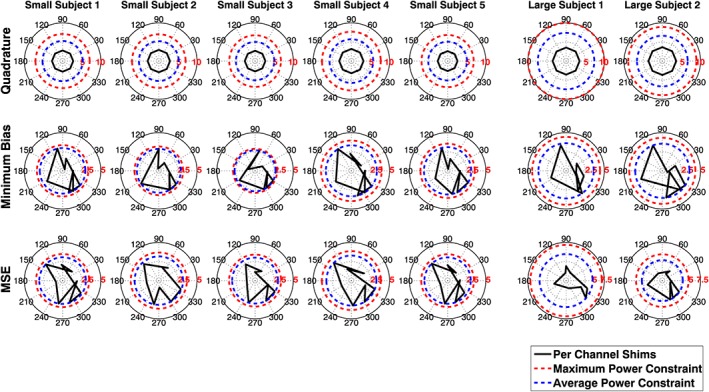
Shim solutions ***w*** for each of the subjects for quadrature, minimum bias and minimum squared error (MSE) methods. The shim values ***w*** are complex and dimensionless – the amplitude corresponds to the relative scaling amplitude. They are plotted here on a polar diagram with phases defined such that quadrature operation is represented as an octagon – equal amplitude on each channel – as for the top row. The maximum and average power constraints are different for each plot because they depend on the pulse duration, TR and *B*_1_^+^ scaling for each subject, according to Equation (17). In the shimmed operating regimes, it is always the average power constraint that is the limiting factor. Quadrature operation is specific absorption rate (SAR) limited, so does not encounter the hardware constraints

**Figure 10:**
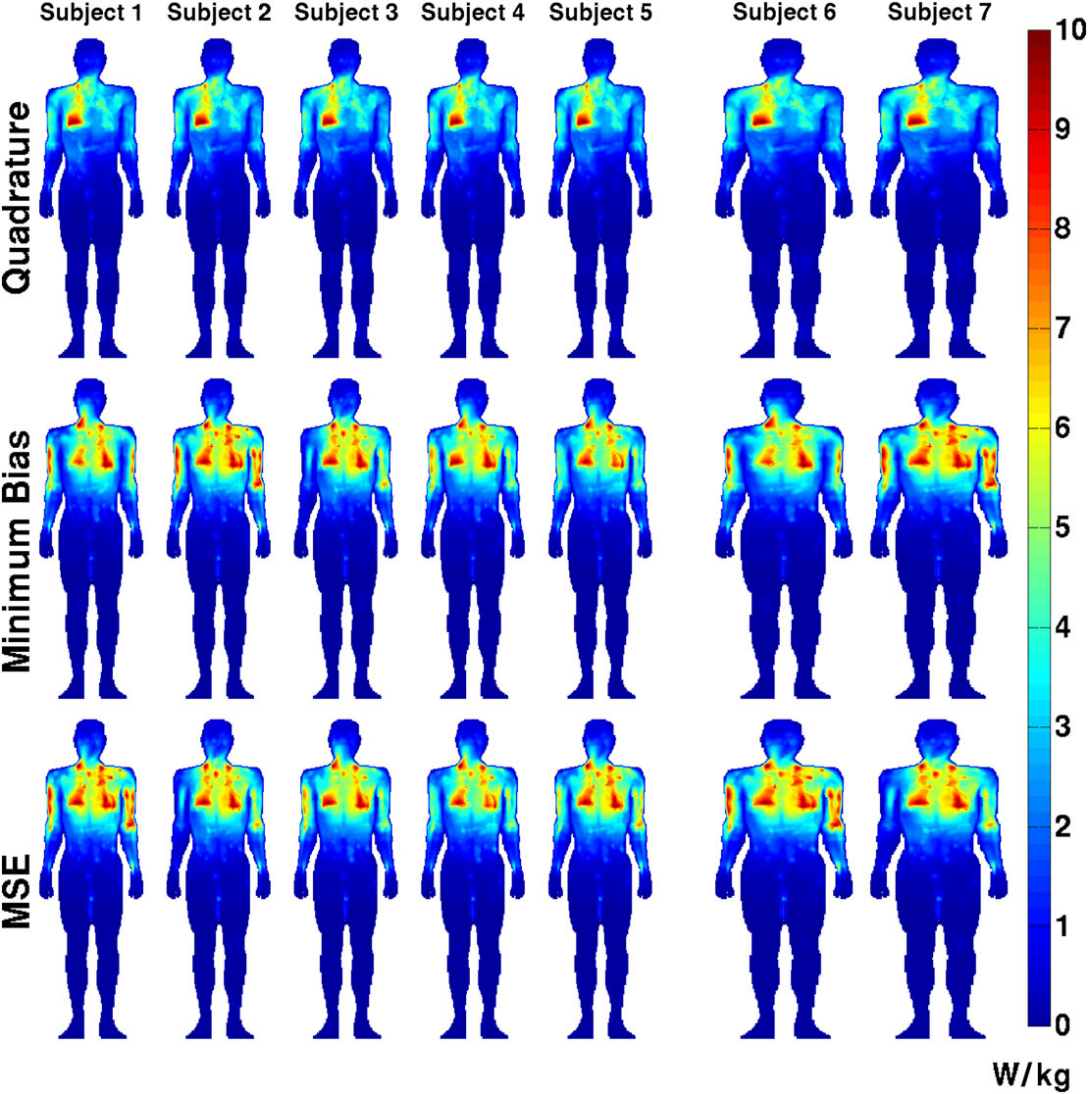
Maximum intensity projections through the voxel models of the specific absorption rate (SAR) in W/kg for each solution. The maximum local SAR is 10 W/kg in all cases. Note that the quadrature solution has an asymmetric SAR distribution which is more uniform for the optimized solutions. The whole‐body SAR is on average 28% higher for the radiofrequency (RF) shimmed solutions compared with quadrature

Optimized RF shim solutions ***w*** are shown in Figure 9. Each one of the optimized solutions has at least one channel that is limited by the average power constraint. It should be noted that the positions of the constraints are different for each plot because each depends on the optimized pulse duration and TR. For the short TR/low pulse amplitude solutions found, the average power constraint is always more limiting. The quadrature mode solutions are also shown for reference; it should be noted that none of these reach hardware limits because they are limited only by SAR, consistent with Figure 2. Figure 10 shows the predicted local SAR distributions for each of the optimized solutions. The maximum local SAR is 10 W/kg for all cases because all sequences were run at the shortest possible TR. The RF shimmed results have more spatially symmetric local SAR distributions that are less strongly peaked at single spatial locations. As a consequence the whole‐body SAR is increased from 0.6 to 0.8 W/kg on average, still below the limit of 2 W/kg (which would have been enforced by the optimization had it applied).

**Figure 11:**
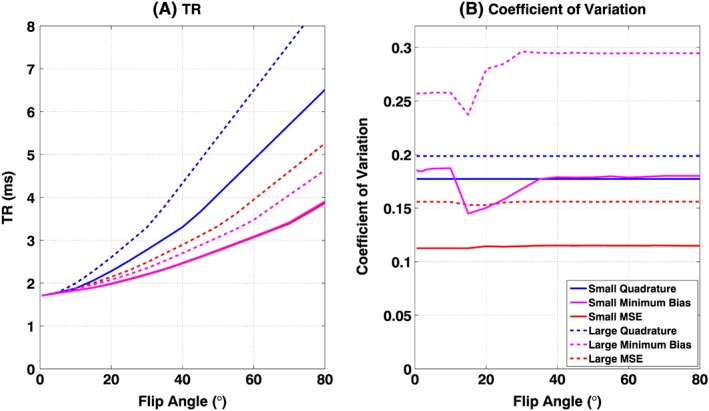
Optimizations for one small (full lines) and one large (broken lines) subject were repeated for multiple target flip angles *θ*_0_. TR (A) and coefficient of variation (B) in *B*_1_^+^ shown for quadrature (blue), minimum bias (magenta) and minimum squared error (MSE) (red). Large reductions in TR are possible for higher target flip angles. The coefficient of variation remains broadly constant for the MSE method, but is less predictable for the minimum bias method

Finally, all *in vivo* examples presented used a target flip angle *θ*_0_ = 45°; however this could in principle be any value. The optimizations were re‐run for two subjects (one small, one large) for a range of target flip angles and the results are summarized in Figure 11. The sequence optimization (whose main objective is to minimize TR) results in substantial reductions in TR, particularly at higher target flip angles. The spatial homogeneity of the achieved flip angle remains relatively constant.

## 4 DISCUSSION

This study has demonstrated the use of PTx RF shimming to optimize at the ‘sequence level’. Other work has focused on the optimization of RF shimming within given (sequence‐specific) parameters.^8^, ^9^ The translation of results from this type of constrained optimization for a given set of hardware and safety constraints to a sequence with other parameters is not straightforward, as the constraints are themselves dependent on sequence parameters, such as RF pulse duration and TR. Instead the proposed approach makes these relationships explicit and explores RF shimming solutions with different sequence properties. The example application used in this work is cardiac MRI with bSSFP, and the overall objective was to minimize the sequence TR whilst holding the flip angle within a desired region of interest (in this case the heart) constant.

The proposed numerical optimization has two stages: an outer stage optimizing the sequence parameters (in this case, RF pulse duration/TR) and an inner stage that computes optimal RF shim settings given these sequence parameters. Two separate inner optimization strategies were explored: one that aimed to achieve the mean desired flip angle within the heart with a 0% bias, and another MSE shim that also improved homogeneity with the trade‐off of 5% bias in the achieved flip angle. The imaging study with seven volunteers using an eight‐channel PTx system (at 3 T) achieved a mean reduction in TR of 1.34 ± 0.28 ms with the MSE shim, whilst simultaneously reducing the coefficient of variation in *B*_1_^+^ within the heart by 33 ± 8% across all subjects, as shown in Figure 6. The minimum bias shim resulted in slightly larger TR reductions at a cost of sometimes worsened homogeneity. The MSE shim was more robust in terms of inter‐subject performance and was always able to produce a substantially improved flip angle distribution in the ROI within the given constraints and allow a significantly reduced TR. The 5% residual bias level was arbitrarily selected as an acceptable trade‐off and corresponds to a mean difference in flip angle of 2°; this could be eliminated by increasing the target flip angle if necessary.

The reduction in TR obtained from RF shimming translated directly into reduced imaging durations and consequently reduced breath‐hold durations for the subjects being imaged. Improved homogeneity and reduced TR are both cited as factors in improving cardiac MRI image quality by other studies.^5^ As well as reducing imaging durations, others have observed that reduced TRs translate to a reduction in severity of banding artifacts and banding‐related flow artifacts in cardiac SSFP imaging.^25^ It should be noted that the reductions in TR are quoted with respect to the quadrature mode sequences that were also optimized using the proposed framework; this was less arbitrary than comparison with a fixed starting sequence which may be suboptimal. Figure 11 shows that optimization for higher flip angles would lead to larger gains in speed when compared with quadrature mode, and the approach could therefore be used when higher flip angles are needed to boost in‐flow contrast.^35^

Increases in scan speed can be attributed to three related effects. The first is that optimization of the sequence parameters leads to a choice of the shortest possible RF pulse duration that can still yield an acceptable RF shimming solution within constraints. Second, optimization of *B*_1_^+^ within a local ROI only around the heart allows RF coil elements further from the heart to be ‘turned down’, therefore reducing their contribution to SAR. This is apparent from the low *B*_1_^+^ in the left and right posterior parts of the torso (Figure 7), and was also noted in van den Bergen et al.^36^ The final related effect is that constrained RF shimming tends to change the local SAR distribution to be more uniform. As a result, the ratio of peak local SAR to whole‐body SAR is reduced, and the scan can then be run faster within the limit – Figure 10 illustrates this effect. It should be noted that whole‐body SAR limits are still respected, and are included in the calculation.

A recent study by Weinberger et al^37^ compared a local four‐channel transmit array with a birdcage coil for 3 T cardiac imaging. The authors limited local SAR to 20 W/kg, arguing that this limit (applying to 10 s of average SAR) is more appropriate for breath‐held cardiac scans than the 6‐min average limit (10 W/kg). Using such a limit with phase‐only shimming, they achieved a TR of 3.8 ms for *θ* = 60° in the heart, with a coefficient of variation of 0.276. The present work used *θ* = 45°; however Figure 11 shows that for a smaller subject, the optimization could achieve TR = 3.1 ms with a coefficient of variation of 0.175 subject to a SAR limit of 10 W/kg for *θ* = 60°. Although the hardware is not identical, the proposed ‘sequence‐level’ approach could potentially lead to even better performance when using the four‐channel coil from Weinberger et al.^37^ Weinberger et al^37^ also discussed the fact that under current IEC guidelines,^2^ the less stringent whole‐body SAR limit of 2 W/kg is the limiting factor for ‘volume RF transmit coils’, whereas the stricter 10 g SAR limit of 10 W/kg applies to ‘local RF transmit coils’. This undoubtedly leads to more conservative operation when using array coils, and also implies that when using a normal body coil, the maximum local SAR is likely to be significantly larger than the limit of 10 W/kg applied in this work. Although the array coil used in this work could potentially be classified as ‘not local’ since it is embedded into the bore of the magnet, the more stringent rules applying to local SAR were employed. The use of less strict SAR constraints could easily be adopted into the presented method, and would lead to improved performance compared with the results presented here.

The preceding discussion also underscores the importance of accurate SAR models. Two different sized SAR models were used, and subjects were selected to correspond either to one or the other of these. Homann et al^7^ showed that the use of larger (higher BMI) models in a constrained optimization always ensured reduced SAR. However such an approach is suboptimal when compared with an appropriate matching model for a given subject. The present study is clearly limited in this sense, as it employs only two separate models and no females – a more extensive set of models, including females, could form the basis for a future study. An alternative approach could be the generation of bespoke EM models^38^; however even in this case validation is difficult. In this work we compared simulated with measured *B*_1_^+^ maps to obtain an approximate measure of validity, as has been performed in a number of previous studies by other groups.^12^, ^39^, ^40^ Doing this in an accurate and quantitative manner is challenging. For this study in particular, comparison of the *B*_1_^+^ maps in the four‐chamber cardiac view is problematic as this is a double oblique view. We found that the mean correlation over all subjects and transmit channels was 0.84 ± 0.04. Homann et al^38^ found that even for bespoke EM models, the standard deviation of differences between model and measurement was on the order of 20%. Errors in SAR models notwithstanding, there is also an ongoing debate over whether temperature rather than SAR should be the constraining factor. This work used SAR as the more conventional approach, but the method could be updated to constrain temperature instead by adopting the ‘T‐matrix’ approach proposed by Boulant et al.^41^

This work used an eight‐channel TEM body coil at 3 T – these types of body array have been used previously for imaging and RF shimming at high field.^42^, ^43^ The coil has elements distributed axially around the magnet bore. This design has good general performance, but is not claimed to be optimal for cardiac MRI. Indeed, the proposed sequence‐level optimization approach is applicable to any PTx coil arrangement. Other work on 3 T cardiac MRI using PTx has focused on local array coils,^44^ and other more general numerical studies have proposed alternative body coil designs that may yield better performance.^45^ The method could also be applied at ultra‐high field (UHF, ≥7 T) where hardware and SAR limits are often more stringent^12^, ^13^, ^46^ and PTx hardware is more common. Several cardiac MRI studies have already been performed at 7 T^47^, ^48^, ^49^, ^50^, ^51^ using local array coils and PTx pulse design methods (‘spokes’ pulses) to improve *B*_1_^+^ homogeneity and robustness to respiration‐induced off‐resonance effects.^52^, ^53^ These are complementary to the present work which focuses instead on the minimization of TR using RF shimming. Simulation studies have indicated that considerable SAR reductions can be achieved using constrained RF shimming for body imaging at UHF.^36^ Different optimal sequence parameters might be expected if sequence‐level optimization is applied to different hardware with different constraints. One practical difference that may arise is the means by which SAR model predictions are normalized to match *in vivo* subjects. In this work, the measured *B*_1_^+^ field in quadrature mode was used; this is convenient for an enveloping array with a well‐defined quadrature (birdcage‐like) mode but may be difficult for a surface array. For localized coils at 7 T, it was found in Restivo et al^54^ that *S*‐parameter measurements can be more appropriate.

An issue for any on‐line optimization method is how it alters the scanning workflow. The acquisition of *B*_1_^+^ maps using DREAM was possible during a single breath‐hold even for eight channels. As noted in the Methods section, DREAM is sensitive to flow effects and the approach taken for excluding these was to manually mask the blood pool on the acquired *B*_1_^+^ maps. Others have approached the same problem by adding flow suppressing pre‐pulses to the sequence.^55^, ^56^ Our results did not show significant flow artifacts in the myocardium, but such a measure could be taken if necessary. A ‘black‐blood’ *B*_1_^+^ mapping sequence would also allow for more straightforward automated segmentation of the myocardium, which would improve the workflow. The larger workflow issue is the computation time, which is currently 3–5 min. Although some steps were taken to reduce this time to an acceptable level, the current work used the CVX software package in Matlab, which is not optimized for speed. Other authors have explored significantly more efficient implementations^12^ which could be adopted to substantially accelerate the inner optimization step. The outer optimization typically requires a small number of iterations; in this study, around 10 were used but it was found that the use of as few as five would have little effect on performance, making a calculation time of under 3 min for the MSE shim feasible. Such an optimization could fit into a workflow if other imaging could be performed during the calculation time, but further computation speed increases are needed before truly real‐time updates are possible.

## 5 CONCLUSION

Sequence‐level application of RF shimming has been proposed as a way of maximizing performance using PTx. The interaction between sequence parameters and typical hardware and safety constraints has been made explicit, allowing optimal constrained RF shimming solutions and optimal pulse sequence operating points to be jointly identified. This is particularly useful for the application focused on in this work – cardiac MRI using bSSFP – since the scanner will typically be running at or close to SAR limits as well as peak or average RF power limits. A study of seven healthy volunteers using an eight‐channel body transmit array at 3 T yielded average TR times of 2.90 ms for a fixed read‐out time of 1.7 ms using MSE optimization, a 31% reduction compared with the equivalently optimized operating point for a single‐channel body coil when running at a local SAR limit of 10 W/kg. The method is not limited to any particular field strength or RF coil design and could be modified to use different pulse sequences, or to consider different optimization targets such as contrast to noise, minimization of SAR or maximization of *B*_1_^+^ homogeneity.

## Supporting information

FIGURE S1. Boxplots of contrast‐to‐noise ratio (CNR) between myocardium and blood within the heart are shown for the images from Figure 5 for all three imaging scenarios. With the extended radiofrequency (RF) shimming regimes, the CNR is increased compared with quadrature, with the minimum squared error (MSE) optimization showing greatest improvement.

## References

[1] Noeske R , Seifert F , Rhein KH , Rinneberg H . Human cardiac imaging at 3 T using phased array coils. Magn Reson Med. 2000;44:978–982. 11108638 10.1002/1522-2594(200012)44:6<978::aid-mrm22>3.0.co;2-9

[2] IEC . Medical electrical equipment. Part 2–33: particular requirements for the safety of magnetic resonance equipment for medical diagnosis. International Electrotechnical Commission; 2015.

[3] Bieri O , Scheffler K . Fundamentals of balanced steady state free precession MRI. J Magn Reson Imaging. 2013;38:2–11. 23633246 10.1002/jmri.24163

[4] Ibrahim TS , Lee R , Baertlein BA , Abduljalil AM , Zhu H , Robitaille PML . Effect of RF coil excitation on field inhomogeneity at ultra high fields: a field optimized TEM resonatior. Magn Reson Imaging. 2001;19:1339–1347. 11804762 10.1016/s0730-725x(01)00404-0

[5] Mueller A , Kouwenhoven M , Naehle C , et al. Dual‐source radiofrequency transmission with patient‐adaptive local radiofrequency shimming for 3.0‐T cardiac MR imaging: initial experience. Radiology. 2012;263:77–86. 22371610 10.1148/radiol.11110347

[6] Krishnamurthy R , Pednekar A , Kouwenhoven M , Cheong B , Muthupillai R . Evaluation of a subject specific dual‐transmit approach for improving B1 field homogeneity in cardiovascular magnetic resonance at 3 T. J Cardiovasc Magn Reson. 2013;15:68 23919374 10.1186/1532-429X-15-68 PMC3750927

[7] Homann H , Graesslin I , Eggers H , et al. Local SAR management by RF shimming: a simulation study with multiple human body models. MAGMA. 2012;25:193–204. 21922191 10.1007/s10334-011-0281-8

[8] Brunner DO , Pruessmann KP . Optimal design of multiple‐channel RF pulses under strict power and SAR constraints. Magn Reson Med. 2010;63:1280–1291. 20432299 10.1002/mrm.22330

[9] Guérin B , Gebhardt M , Cauley S , Adalsteinsson E , Wald LL . Local specific absorption rate (SAR), global SAR, transmitter power, and excitation accuracy trade‐offs in low flip‐angle parallel transmit pulse design. Magn Reson Med. 2014;71:1446–1457. 23776100 10.1002/mrm.24800 PMC3871989

[10] Van Den Berg CAT , Van Den Bergen B , Van Den Kamer JB , et al. Simultaneous B1+ homogenization and specific absorption rate hotspot suppression using a magnetic resonance phased array transmit coil. Magn Reson Med. 2007;57:577–586. 17326185 10.1002/mrm.21149

[11] Lee J , Gebhardt M , Wald LL , Adalsteinsson E . Local SAR in parallel transmission pulse design. Magn Reson Med. 2012;67:1566–1578. 22083594 10.1002/mrm.23140 PMC3291736

[12] Hoyos‐Idrobo A , Weiss P , Massire A , Amadon A , Boulant N . On variant strategies to solve the magnitude least squares optimization problem in parallel transmission pulse design and under strict SAR and power constraints. IEEE Trans Med Imaging. 2014;33:739–748. 24595346 10.1109/TMI.2013.2295465

[13] Snyder CJ , Delabarre L , Moeller S , et al. Comparison between eight‐ and sixteen‐channel TEM transceive arrays for body imaging at 7 T. Magn Reson Med. 2012;67:954–964. 22102483 10.1002/mrm.23070 PMC3290686

[14] Graesslin I , Homann H , Biederer S , et al. A specific absorption rate prediction concept for parallel transmission MR. Magn Reson Med. 2012;68:1664–1674. 22231647 10.1002/mrm.24138

[15] Homann H , Graesslin I . Specific absorption rate reduction in parallel transmission by k‐space adaptive radiofrequency pulse design. Magn Reson Med. 2011;357:350–357. 10.1002/mrm.22663 21264927

[16] Eichfelder G , Gebhardt M . Local specific absorption rate control for parallel transmission by virtual observation points. Magn Reson Med. 2011;66:1468–1476. 21604294 10.1002/mrm.22927

[17] Vernickel P , Röschmann P , Findeklee C , et al. Eight‐channel transmit/receive body MRI coil at 3 T. Magn Reson Med. 2007;58:381–389. 17654592 10.1002/mrm.21294

[18] Wackerly DD , Mendenhall W , Scheaffer RL . Mathematical Statistics with Applications. 7th ed. Belmont, California: Thomson Brooks/Cole; 2008.

[19] Wang C , Shen GX . B1 field, SAR, and SNR comparisons for birdcage, TEM, and microstrip coils at 7 T. J Magn Reson Imaging. 2006;24:439–443. 16786582 10.1002/jmri.20635

[20] Clemens M , Weil T . Discrete electromagnetism with the finite integration technique. Prog Electromagn Res. 2001;32:65–87.

[21] Kozlov M , Turner R . Fast MRI coil analysis based on 3‐D electromagnetic and RF circuit co‐simulation. J Magn Reson. 2009;200:147–152. 19570700 10.1016/j.jmr.2009.06.005

[22] Beqiri A , Hand JW , Hajnal JV , Malik SJ . Comparison between simulated decoupling regimes for specific absorption rate prediction in parallel transmit MRI. Magn Reson Med. 2015;74:1423–1434. 25367780 10.1002/mrm.25504 PMC7613953

[23] Dimbylow PJ . FDTD calculations of the whole‐body averaged SAR in an anatomically realistic voxel model of the human body from 1 MHz to 1 GHz. Phys Med Biol. 1997;42:479–490. 9080530 10.1088/0031-9155/42/3/003

[24] Jin J , Liu F , Weber E , Crozier S . Improving SAR estimations in MRI using subject‐specific models. Phys Med Biol. 2012;57:8153–8171. 23174940 10.1088/0031-9155/57/24/8153

[25] Schär M , Kozerke S , Fischer SE , Boesiger P . Cardiac SSFP imaging at 3 Tesla. Magn Reson Med. 2004;51:799–806. 15065254 10.1002/mrm.20024

[26] Nehrke K . Börnert P. DREAM—a novel approach for robust, ultrafast, multislice B1 mapping. Magn Reson Med. 2012;68:1517–1526. 22252850 10.1002/mrm.24158

[27] Brunner D , Pruessmann K . A matrix approach for mapping array transmit fields in under a minute. *Proceedings of the 16th Annual Meeting ISMRM*, Toronto, ON, Canada, 2008; 354.

[28] Lagarias JC , Reeds JA , Wright MH , Wright PE . Convergence properties of the Nelder–Mead simplex method in low dimensions. SIAM J Optim. 1998;9:112–147.

[29] Grant M , Boyd S . Graph implementations for nonsmooth convex programs In: Blondel V, Boyd S, Kimura H, eds. Recent Advances in Learning and Control. Lecture Notes in Control and Information Sciences. Springer‐Verlag; 2008:95–110.

[30] Grant M , Boyd S . {CVX}: Matlab Software for Disciplined Convex Programming, version 2.1.

[31] Sturm JF . SeDuMi – Software for Optimization over Symmetric Cones.

[32] Setsompop K , Wald LL , Alagappan V , Gagoski BA , Adalsteinsson E . Magnitude least squares optimization for parallel radio frequency excitation design demonstrated at 7 Tesla with eight channels. Magn Reson Med. 2008;59:908–915. 18383281 10.1002/mrm.21513 PMC2715966

[33] Kassakian PW . Convex Approximation and Optimization with Applications in Magnitude Filter Design and Radiation Pattern Synthesis. Berkeley, CA: University of California at Berkeley; 2006.

[34] Boyd S , Vandenberghe L . Convex Optimization. Cambridge: Cambridge University Press; 2004.

[35] Markl M , Pelc NJ . On flow effects in balanced steady‐state free precession imaging: pictorial description, parameter dependence, and clinical implications. J Magn Reson Imaging. 2004;20:697–705. 15390233 10.1002/jmri.20163

[36] van den Bergen B , Van den Berg CAT , Bartels LW , Lagendijk JJW . 7 T body MRI: B1 shimming with simultaneous SAR reduction. Phys Med Biol. 2007;52:5429–5441. 17762096 10.1088/0031-9155/52/17/022

[37] Weinberger O , Winter L , Dieringer MA , et al. Local multi‐channel RF surface coil versus body RF coil transmission for cardiac magnetic resonance at 3 Tesla: which configuration is winning the game? PLoS One. 2016;11:e0161863 27598923 10.1371/journal.pone.0161863 PMC5012568

[38] Homann H , Börnert P , Eggers H , Nehrke K , Dössel O , Graesslin I . Toward individualized SAR models and in vivo validation. Magn Reson Med. 2011;66:1767–1776. 21630346 10.1002/mrm.22948

[39] Hoffmann J , Shajan G , Scheffler K , Pohmann R . Numerical and experimental evaluation of RF shimming in the human brain at 9.4 T using a dual‐row transmit array. MAGMA. 2014;27:373–386. 24276542 10.1007/s10334-013-0419-y

[40] Van den Berg CAT , Bartels LW , van den Bergen B , et al. The use of MR B + 1 imaging for validation of FDTD electromagnetic simulations of human anatomies. Phys Med Biol. 2006;51:4735–4746. 16985267 10.1088/0031-9155/51/19/001

[41] Boulant N , Wu X , Adriany G , Schmitter S , Uğurbil K , Van de Moortele PF . Direct control of the temperature rise in parallel transmission by means of temperature virtual observation points: simulations at 10.5 tesla. Magn Reson Med. 2015;256:249–256. 10.1002/mrm.25637 PMC4561040 25754685

[42] Vaughan JT , Adriany G , Snyder CJ , et al. Efficient high‐frequency body coil for high‐field MRI. Magn Reson Med. 2004;52:851–859. 15389967 10.1002/mrm.20177

[43] Vaughan JT , Hetherington HP , Otu JO , Pan JW , Pohost GM . High frequency volume coils for clinical NMR imaging and spectroscopy. Magn Reson Med. 1994;32:206–218. 7968443 10.1002/mrm.1910320209

[44] Kraus O , Lukas W , Matthias D , et al. Local coil versus conventional body coil transmission for cardiac MR: B1+ efficiency improvements and enhanced blood myocardium. *Proceedings of the XXth Annual Meeting ISMRM*, 22nd, Milan, Italy, 2014;2364:

[45] Guérin B , Gebhardt M , Serano P , et al. Comparison of simulated parallel transmit body arrays at 3 T using excitation uniformity, global SAR, local SAR, and power efficiency metrics. Magn Reson Med. 2014;00:1–14. 10.1002/mrm.25243 PMC4201892 24752979

[46] Deniz CM , Alon L , Brown R , Zhu Y . Subject‐ and resource‐specific monitoring and proactive management of parallel radiofrequency transmission. Magn Reson Med. 2016;76(1):20–31. 26198052 10.1002/mrm.25828 PMC4721949

[47] Suttie JJ , Delabarre L , Pitcher A , et al. 7 Tesla (T) human cardiovascular magnetic resonance imaging using FLASH and SSFP to assess cardiac function: validation against 1.5 T and 3 T. NMR Biomed. 2012;25:27–34. 21774009 10.1002/nbm.1708 PMC3440016

[48] Snyder CJ , DelaBarre L , Metzger GJ , et al. Initial results of cardiac imaging at 7 Tesla. Magn Reson Med. 2009;61:517–524. 19097233 10.1002/mrm.21895 PMC2939145

[49] Thalhammer C , Renz W , Winter L . Two‐dimensional sixteen channel transmit/receive coil array for cardiac MRI at 7.0 T: design, evaluation, and application. J Magn Reson Imaging. 2012;36:847–857. 22706727 10.1002/jmri.23724 PMC3445730

[50] Graessl A , Renz W , Hezel F , et al. Modular 32‐channel transceiver coil array for cardiac MRI at 7.0 T. Magn Reson Med. doi: 10.1002/mrm.24903 10.1002/mrm.24903 23904404

[51] Maderwald S , Orzada S , Schäfer L , et al. 7 T human in vivo cardiac imaging with an 8‐channel transmit/receive array. *Proceedings of the 17th Annual Meeting ISMRM*, Honolulu, HI, 2009;17:2716.

[52] Schmitter S , Delabarre L , Wu X , et al. Cardiac imaging at 7 tesla: single‐ and two‐spoke radiofrequency pulse design with 16‐channel parallel excitation. Magn Reson Med. 2013;70:1210–1219. 24038314 10.1002/mrm.24935 PMC3960017

[53] Schmitter S , Wu X , Ugurbil K , Van De Moortele PF . Design of parallel transmission radiofrequency pulses robust against respiration in cardiac MRI at 7 Tesla. Magn Reson Med. 2015;74:1291–1305. 25411131 10.1002/mrm.25512 PMC4437923

[54] Restivo M , Raaijmakers A , Van Den Berg C , Luijten P , Hoogduin H . Improving peak local SAR prediction in parallel transmit using in situ S‐matrix measurements. Magn Reson Med. 2016;00:1–8. 10.1002/mrm.26261 27173968

[55] Nehrke K , Sprinkart AM , Schild HH , Börnert P . Fast B1+ mapping for cardiac MR using a black blood DREAM sequence. *Proceedings of the 21st ISMRM*, Salt Lake City, 2013;4271.

[56] Börnert P , Nehrke K , Wang J . Magnetization prepared DREAM for fast flow‐robust B1+ mapping. *Proceedings of the 22nd ISMRM*, Milan, 2014;22:4338.

